# Vector Competence of *Aedes aegypti, Aedes albopictus* and *Culex quinquefasciatus* from Brazil and New Caledonia for Three Zika Virus Lineages

**DOI:** 10.3390/pathogens9070575

**Published:** 2020-07-16

**Authors:** Rosilainy S. Fernandes, Olivia O’Connor, Maria Ignez L. Bersot, Dominique Girault, Marguerite R. Dokunengo, Nicolas Pocquet, Myrielle Dupont-Rouzeyrol, Ricardo Lourenço-de-Oliveira

**Affiliations:** 1 Laboratório de Mosquitos Transmissores de Hematozoários, Instituto Oswaldo Cruz-FIOCRUZ, Rio de Janeiro 21040-900, Brazil; rosilainysf@gmail.com (R.S.F.); ignez@ioc.fiocruz.br (M.I.L.B.); 2 Institut Pasteur de Nouvelle-Calédonie, URE Dengue et Arboviroses, Réseau International des Instituts Pasteur, 98800 Noumea, New Caledonia; ooconnor@pasteur.nc (O.O.); dgirault@pasteur.nc (D.G.); rowynadokunengo@outlook.fr (M.R.D.); 3 Institut Pasteur de Nouvelle-Calédonie, URE Entomologie Médicale, Réseau International des Instituts Pasteur, 98800 Noumea, New Caledonia; npocquet@pasteur.nc

**Keywords:** transmission efficiency, vector capacity, susceptibility

## Abstract

Zika virus (ZIKV) has caused severe epidemics in South America beginning in 2015, following its spread through the Pacific. We comparatively assessed the vector competence of ten populations of *Aedes* *aegypti* and *Ae. albopictus* from Brazil and two of *Ae.* *aegypti* and one of *Culex quinquefasciatus* from New Caledonia to transmit three ZIKV isolates belonging to African, Asian and American lineages. Recently colonized mosquitoes from eight distinct sites from both countries were orally challenged with the same viral load (10^7^ TCID_50_/mL) and examined after 7, 14 and 21 days. *Cx. quinquefasciatus* was refractory to infection with all virus strains. In contrast, although competence varied with geographical origin, Brazilian and New Caledonian *Ae. aegypti* could transmit the three ZIKV lineages, with a strong advantage for the African lineage (the only one reaching saliva one-week after challenge). Brazilian *Ae. albopictus* populations were less competent than *Ae. aegypti* populations. *Ae. albopictus* generally exhibited almost no transmission for Asian and American lineages, but was efficient in transmitting the African ZIKV. Viral surveillance and mosquito control measures must be strengthened to avoid the spread of new ZIKV lineages and minimize the transmission of viruses currently circulating.

## 1. Introduction

Zika virus (ZIKV) is an arbovirus (Flaviviridae, Flavivirus) that originated in Africa, where it is transmitted either in the wild or modified environments by *Aedes* mosquitoes. The virus was little known for many decades after its discovery in the 1940s. However, beginning in 2007, when it caused the first outbreak detected outside Africa (Yap Island, Federated States of Micronesia, Pacific region) the virus gained notoriety [1,2]. In 2013, ZIKV emerged in French Polynesia [3], leading to more than 8700 suspected cases and 30,000 medical consultations reported by the sentinel surveillance network [4]. A recent seroprevalence study estimated that more than half of the population was infected by ZIKV in French Polynesia [5]. From French Polynesia, ZIKV spread to New Caledonia in 2013 [6,7], affecting the whole territory. The New Caledonia Health Authorities estimated the number of cases at about 11,000 [8]. From 2014 to 2017, ZIKV was detected in the Cook Islands, Easter Islands, Vanuatu, Fiji, Samoa, Solomon Islands, Tonga and American Samoa [8,9]. During the same period, ZIKV spread to the American continent, being first detected in northeastern Brazil in 2015 [10]. Subsequently, countrywide epidemics were reported in Brazil as well as several South and Central American localities. In 2015, Brazil reported 37,011 probable Zika symptomatic infections and the first cases of microcephaly associated with ZIKV [10,11]. The number of cases in Brazil peaked in 2016 with nearly 215,320 probable Zika cases [12], followed by 17,593 and 10,768 reported cases annually between 2017 and 2019 [13,14]. The occurrence of microcephaly and other congenital neurological malformations associated with ZIKV infections has been described worldwide [15,16,17], and the virus continues to be considered an important threat [18].

Although ZIKV can be directly transmitted between humans, vector borne transmission is believed to play a major role in virus spread [19,20]. *Aedes aegypti* is generally considered the primary vector of ZIKV in all surveyed areas [20,21,22]. Substantial variation in vector competence, however, has been described in populations of *Ae. aegypti* of different geographical origins when challenged with distinct ZIKV strains controlling for titer in infectious blood meal [20,23]. In ZIKV epidemic and endemic areas, *Ae. aegypti* cooccurs with other domestic and peridomestic mosquitoes such as *Culex quinquefasciatus* and *Aedes albopictus* that frequently bite humans. Natural ZIKV infections have been reported in these species, and thus, they came under suspicion as alternative vectors [24,25,26,27]. Concerning *Ae. albopictus*, results of vector competence evaluations to date have shown that this mosquito is susceptible to ZIKV but with a transmission efficiency that is significantly inferior to that of *Ae. aegypti* [20,25]. In contrast, domestic *Culex* species tested worldwide to date have consistently been shown to be essentially refractory to ZIKV, and thus, their potential role in ZIKV transmission has become controversial [28,29,30,31].

Phylogenetic studies have shown that Brazilian ZIKV isolates clustered with other American isolates in the so-called American lineage, sharing common ancestry with the Asian genotype that includes strains that circulated in French Polynesia and other Pacific areas such as New Caledonia in 2013–2014 [32,33,34]. Interestingly, *Ae*. *aegypti* and *Ae*. *albopictus* from Brazil exhibited lower vector competence for a ZIKV from New Caledonia than for isolates from Brazil [28,35]. And curiously, *Ae*. *aegypti* from Singapore was also less competent in experimentally transmitting a French Polynesian isolate than a Brazilian one [36].

It is clear that vector competence is dependent on the specific combination of mosquito and virus genotypes from different geographic regions [37,38]. Determining vector competence of human biting mosquito populations from endemic ZIKV territories is required to inform control measures. Here, we comparatively assessed the vector competence of 13 mosquito populations of *Ae. aegypti, Ae. albopictus* and *Cx. quinquefasciatus* from New Caledonia and Brazil to African, Asian and American ZIKV strains.

## 2. Results

### 2.1. Aedes aegypti from All Brazilian Regions Can Experimentally Transmit ZIKV Isolated from Africa, Asia and America, but with Different Competence

**Figure 1:**
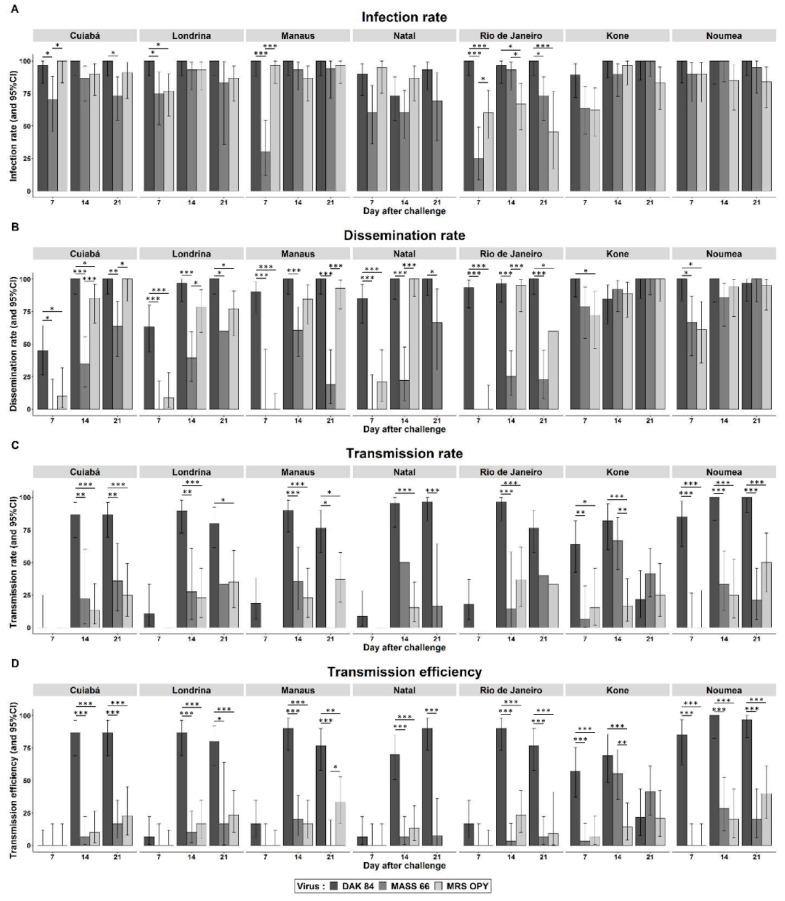
Vector competence results for *Aedes aegypti* from different cities of Brazil (Cuiabá,Londrina, Manaus, Natal and Rio de Janeiro) and New Caledonia (Kone and Noumea) orally challenged with three ZIKV isolates: DAK 84 (African lineage), MASS 66 (Asian lineage), MRS OPY (American lineage). (**A**) Infection rate, (**B**) dissemination rate, (**C**) transmission rate and (**D**) transmission efficiency at 7, 14 and 21 days after challenge. Error bars represent 95% confidence intervals. Significant differences are indicated by asterisks (Fisher’s Exact test: * *p* < 0.05; ** *p* < 0.01; *** *p* < 0.001).

Five Brazilian populations of *Ae. aegypti* were orally challenged with three strains of ZIKV; the population represented the five geographic regions of the country. While infection was detected in mosquitoes of all populations, regardless of the incubation time and the virus tested (Figure 1A, Table S1), quantitative infection rates (IR) varied depending on the virus strain and the incubation time. In general, IR did not increase with incubation time, regardless of the tested ZIKV isolates; the results from the African isolate were a very representative example of this trend (Figure 1). Except for the population of Natal, the IRs with the African genotype were always above 90%, being 100% for most populations, regardless of the incubation time. There was greater heterogeneity in the IR with the different viruses at 7 d.p.i. than at 14 and 21 d.p.i., except for the population of Rio de Janeiro, where significant differences were detected between essentially all viruses in all incubation times (Figure 1A). At 7 d.p.i., the IRs were significantly lower for the ZIKV isolate of the Asian lineage in all challenged Brazilian *Ae. aegypti* except for Natal. In general, IRs tended to be similar among viruses at 14 d.p.i., except for the Rio de Janeiro *Ae. aegypti* population.

All three viruses tested were able to disseminate to secondary tissues (head) in all challenged Brazilian *Ae. aegypti* populations, albeit to varying extents (Figure 1B, Table S1). However, in contrast to IR, the dissemination rates (DR) were quite heterogeneous among the tested viruses and Brazilian populations. At 7 d.p.i., DR were generally null or nonsignificant for all mosquito populations challenged with the two ZIKV isolates of the Asian genotype. In contrast, at this same incubation time, the African genotype isolate had already surprisingly disseminated to the heads of more than 80% of *Ae. aegypti* from Rio de Janeiro, Manaus and Natal. Albeit with lower rates, the African genotype also disseminated in significantly higher rates and more rapidly than the other viruses in the mosquitoes of Londrina and Cuiaba. At 14 d.p.i., all three tested viruses disseminated to the heads of mosquitoes of all five Brazilian populations, but with heterogeneous DR. Again, regardless of the mosquito population, the DRs of the Asian lineage were lower than those detected for African and American lineages at 14 and 21 d.p.i. (Figure 1B). In general, from two weeks after the infectious meal, these two viral isolates disseminated in more than 75% of infected mosquitoes. The DR values increased over the incubation time (*p* < 0.05) in the Cuiabá and Londrina *Ae. aegypti* infected with any of the ZIKV isolates and in the Manaus and Natal populations infected with the Asian and American lineages (Tables S3–S5).

**Figure 2:**
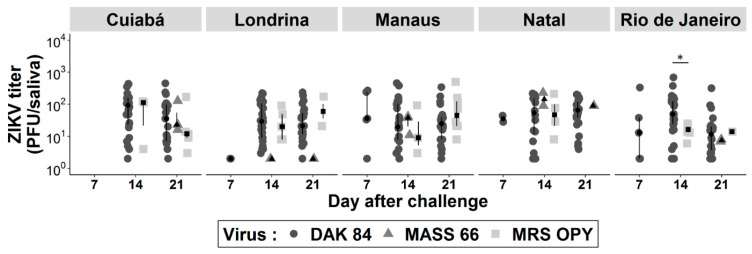
ZIKV load in saliva of *Ae*. *aegypti* from five Brazilian cities at 7, 14 and 21 days after oral challenge with three isolates: DAK 84 (African lineage), MASS 66 (Asian lineage), MRS OPY (American lineage). Virus was detected by plaque forming unit (PFU) assays on Vero cells. Significant difference is indicated by asterisk (Wilcoxon test: * *p* < 0.05).

Interestingly, from 14 d.p.i. on, transmission was achieved in all combinations of Brazilian *Ae. aegypti* and virus lineages, except for the isolate belonging to the African lineage which was transmitted as early as 7 d.p.i. by four out of the five challenged populations (Figure 1C and Figure 2D, Table S1). It is noteworthy that from 14 d.p.i. on, infectious particles of the ZIKV of the African genotype were detected in over 75% of individuals from all five mosquito populations in which the virus had disseminated, and the transmission rates (i.e., the proportion of mosquitoes with infectious viral particles in saliva among those with disseminated infection, or TR) increased with incubation time (*p* < 0.05). In general, transmission efficiency values (the proportion of mosquitoes with virus in saliva among all orally challenged mosquitoes, or TE) were higher in the African lineage compared to the other two lineages. With few exceptions (TE for Manaus, Londrina and Rio de Janeiro; *p* < 0.05), there was no significant difference between the TRs or TEs of the two ZIKV isolates of the Asian and American lineages in any population of *Ae. aegypti* from Brazil.

The median viral load in Brazilian *Ae. aegypti* saliva ranged from 2 to 36, 2 to 160.5 and 2 to 97 PFU at 7, 14 and 21 d.p.i., respectively (Table S2). The viral load per saliva sample reached values as high as 685, 502 and 230 PFU when infected with the African, American and Asian lineage, respectively. No difference was found when comparing viral load in positive saliva expectorated by *Ae. aegypti* individuals belonging to the same population infected with the three different viruses, regardless of incubation time, except for those from Rio de Janeiro infected with the African genotype compared to the American lineage at 14 d.p.i. (*p* = 0.02) (Figure 2).

Comparing *Ae. aegypti* populations, only the viral titers in the saliva of mosquitoes from Natal were higher than those from Rio de Janeiro (*p* = 0.001) infected with the African ZIKV at 21 d.p.i, whereas the pairwise comparisons with the American and Asian lineages and incubation times did not differ (Tables S3–S5).

### 2.2. Two Populations of Aedes aegypti from New Caledonia Can Experimentally Transmit ZIKV Isolated from Africa, Asia and America, but with Different Competence

Two New Caledonian populations of *Ae. aegypti* were orally challenged with three strains of ZIKV. Infection was detected in mosquitoes of both populations, regardless of the incubation time and the virus tested (Figure 1A, Table S1). In general, IR was already high at 7 d.p.i, and did not increase with incubation time with any of the three ZIKV isolates. The IRs with the African genotype were always above 85%, regardless of the incubation time. Some heterogeneity between the three ZIKV strains could be observed with *Ae. aegypti* from Kone, with the IR ranging from 62% to 89% at 7 d.p.i.

All three viruses tested were able to disseminate to secondary tissues in both New Caledonian *Ae. aegypti* populations (Figure 1B, Table S1). The DRs increased over time and ranged from 61 to 100% across the three virus isolates and two New Caledonian mosquito populations. Only at 7 d.p.i., a significantly higher DR for the African ZIKV genotype was observed compared to the Asian genotype for both *Ae. aegypti* populations (*p* = 0.01 for Kone, and *p* = 0.01 and =0.01 for Noumea, compared to ZIKV American and Asian lineages respectively).

Transmission was achieved in all combinations of New Caledonian *Ae. aegypti* and virus lineages assayed from 14 d.p.i. on, except for the isolate belonging to the African genotype, which was transmitted as early as 7 d.p.i. (Figure 1C,D, Table S1). For the African genotype, TR increased over time for both *Ae. aegypti* populations (57 to 82%) except for *Ae. aegypti* from Kone at 21 d.p.i, with a lower level of infectious saliva detected in individuals (17%). In general, the African genotype was also by far the most efficiently transmitted for both *Ae. aegypti* populations compared to the Asian one (*p* < 0.001). There was no significant difference between the TR or TE of the two ZIKV isolates of the Asian genotype in any population of *Ae. aegypti* from New Caledonia, except at 14 d.p.i. for Kone (*p* < 0.01).

Comparing the two New Caledonian *Ae. aegypti* populations, no significant differences were detected for IR and DR, irrespective of the d.p.i. and the ZIKV strains. Regarding the TR, *Ae. aegypti* from Noumea was higher at 21 d.p.i. for the African genotype compared to *Ae. aegypti* from Kone (*p* < 0.001). Likewise, the TE of African genotype was higher for *Ae. aegypti* from Noumea compared to those from Kone at 21 d.p.i. (*p* < 0.001). Unfortunately, due to technical issues, it was not possible to estimate the viral load in the saliva of New Caledonia *Ae. aegypti*.

### 2.3. Aedes aegypti from Brazil and New Caledonia Have Similar Transmission Efficiency for ZIKV of the American Lineage, but Differ Regarding the African and Asian Lineages at Some Incubation Periods

In comparisons of *Ae. aegypti* populations, no significant differences (*p* > 0.05) were found between the TR and TE values exhibited by the seven Brazilian and two New Caledonian *Ae. aegypti* populations challenged with ZKV of the American lineage, irrespective of incubation time (Tables S3–S5). On the other hand, some significant differences were found in TR and/or TE with the African and Asian ZIKV lineages. Interestingly, with the African ZIKV isolate, TR and TE values at 7 d.p.i were significantly much higher for both New Caledonian populations (Noumea: *p* < 0.001 and Kone: *p* < 0.05) when compared with any Brazilian *Ae. aegypti*. However, at 14 d.p.i., TR and TE values were statistically similar (*p* > 0.05) for all comparisons with the African isolate. At 21 d.p.i, TR and TE values were also essentially similar for all comparisons, with one exception: the New Caledonian *Ae. aegypti* from Kone were significantly much less efficient (*p* < 0.01) in transmitting the African isolate than all other tested populations. When considering transmission of the Asian lineage, TR values did not differ in any pairwise comparisons at 7, 14 and 21 d.p.i. In contrast, when considering TE at 14 d.p.i., values for the Asian lineage were much higher (*p* < 0.01) for the New Caledonian Kone population compared to all Brazilian ones except Manaus. At 21 d.p.i., TE values for Kone continued to be lower than Manaus, Rio (*p* < 0.05) when challenged with ZIKV of the Asian lineage.

### 2.4. Brazilian Ae. albopictus Poorly Transmits ZIKV Isolates of the Asian Genotype Compared to the African One

**Figure 3:**
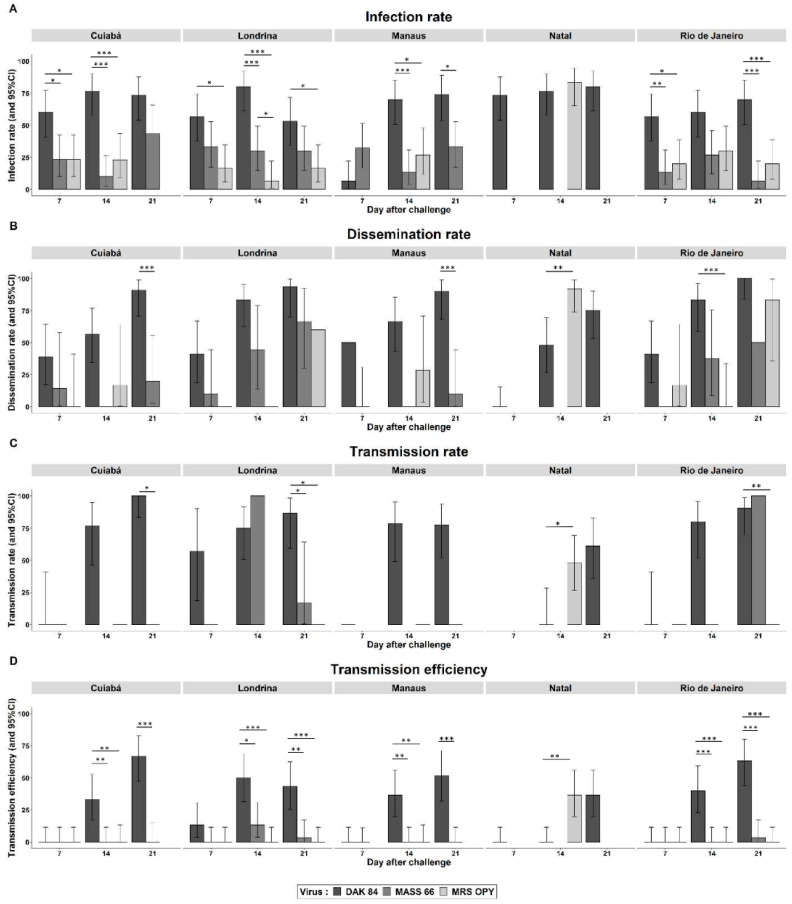
Vector competence results for *Aedes albopictus* from different Brazilian cities orally challenged with three ZIKV three isolates: DAK 84 (African lineage), MASS 66 (Asian lineage), MRS OPY (American lineage). (**A**) Infection rate, (**B**) dissemination rate, (**C**) transmission rate and (**D**) transmission efficiency at 7, 14 and 21 days after oral challenge. Error bars represent 95% confidence intervals. Significant differences are indicated by asterisks (Fisher’s Exact test: * *p* < 0.05; ** *p* < 0.01; *** *p* < 0.001).

We assessed vector competence for the same three strains of ZIKV in five Brazilian populations of *Ae. albopictus* sympatric to the tested *Ae. aegypti* populations. All orally challenged *Ae. albopictus* populations became infected with the three virus isolates, but with significantly higher IR for the African lineage compared to the Asian and American ones (Figure 3A, Table S1). This difference is quite evident for all tested *Ae. albopictus* populations, except for that of Natal, where no significant differences were found between IRs with the American and African isolates at 14 d.p.i. As for the viruses of the Asian ZIKV genotype, the population of Natal could not be challenged with the Asian lineage due to an insufficient number of mosquitoes.

All three ZIKVs disseminated in all *Ae. albopictus* populations at some time point (Figure 3B, Table S1). Values of DRs significatively increased over time (*p* < 0.05) in all *Ae. albopictus* populations infected with the African ZIKV, except in the mosquitoes from Manaus. No difference in DR values was detected at 7 d.p.i. At 14 or 21 d.p.i., the African virus disseminated at a significantly higher rate than the isolates of the Asian genotype, except for the Londrina and Natal populations. The ZIKV isolate of the American lineage took longer than the other lineages to disseminate to secondary tissues in *Ae. albopictus* from Cuiabá, Manaus (14 d.p.i.) and Londrina (21 d.p.i.).

Transmission was detected from 14 d.p.i. in all tested *Ae. albopictus* populations. With the population of Londrina infected with the African virus, infective particles were detected in saliva even earlier, i.e., at 7 d.p.i. (Figure 3C,D, Table S1). Only the African ZIKV was transmitted by all *Ae. albopictus* populations. Transmission of the Asian and American lineages was heterogeneous among *Ae. albopictus,* and very low to null TR and TE were usually recorded. The populations of Cuiabá and Manaus were unable to transmit any virus of the Asian genotype, while those from Londrina and Rio de Janeiro were able to transmit only the Asian isolate but not the American one; the latter isolate was transmitted by Natal *Ae. albopictus.* When considering the initial number of challenged *Ae. albopictus,* TE values were zero to less than 20% for almost all combinations of mosquito population, virus isolate and incubation time, with a few exceptions with the African genotype. For instance, transmission was detected in ~70% of *Ae. albopictus* from Rio de Janeiro and Cuiabá challenged with ZIKV of the African genotype.

**Figure 4:**
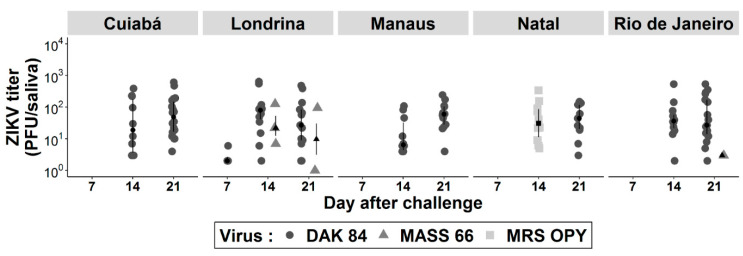
ZIKV load in saliva of *Ae*. *albopictus* from five Brazilian cities at 7, 14 and 21 days after oral challenge with three isolates: DAK 84 (African lineage), MASS 66 (Asian lineage), MRS OPY (American lineage). Virus was detected by plaque forming unit (PFU) assays on Vero cells.

No difference was detected between viral load in saliva expectorated by Brazilian *Ae. albopictus* of the same population infected with the three different isolates (Figure 4). The maximum viral load per saliva of *Ae. albopictus* were 638, 337 and 127 PFU when infected with the African, American and Asian lineages, respectively. Among all *Ae. albopictus* saliva samples at 7 d.p.i, the only positive ones were from four individuals from Londrina with a median of only 2 PFU of ZIKV of the African genotype. The median viral loads varied from 6.5 to 80 and 3 to 60 PFU at 14 and 21 d.p.i., respectively (Table S6).

When comparing viral load per positive saliva between populations of Brazilian *Ae. albopictus* challenged with the same isolate, no significant difference was detected (Tables S3–S5).

### 2.5. Brazilian Ae. aegypti Are Superior in Vector Competence to All Tested ZIKV Compared to Sympatric Ae. albopictus

The comparison of vector competence of sympatric *Aedes* species from the five Brazilian localities revealed that *Ae. aegypti* is generally much more permissive than *Ae. albopictus* for infection with all three ZIKV lineages. The IR values for *Ae. aegypti* were significantly higher than for sympatric *Ae. albopictus* challenged with the same ZIKV isolate and incubation period in 62% of the 37 possible pairwise comparisons; differences were expressively high (*p* < 0.001 or <0.01) in 15 out of the 37 comparisons (see Table S1). In general, the IRs for Londrina Cuiabá, Manaus and Rio de Janeiro *Ae. aegypti* were significantly higher than for sympatric *Ae. albopictus* despite the ZIKV isolates and incubation times (Tables S3–S5). Curiously, no difference in IR (*p* > 0.05) was found between mosquito species from Natal, although the number of possible comparisons in this case is relatively small. There was a clear tendency for a superiority of *Ae. aegypti* compared to sympatric Brazilian *Ae. albopictus* in the dissemination and transmission of the tested virus, although we were unable to demonstrate this trend statistically in most cases due to the sample size. In fact, the number of individuals with infectious heads among infected mosquitoes, and of individuals with infectious saliva among those with disseminated infection, in *Ae. albopictus* populations was extremely low or nonexistent, greatly decreasing the usefulness of making interspecies comparisons. Despite this limitation, significant differences (*p* < 0.05) in DR and TE between sympatric *Ae. aegypti* and *Ae. albopictus* could be demonstrated, specially concerning the challenges with the African ZIKV isolate, and to a lesser extent, with the American lineage (Table S1). Despite being heterogeneous, all populations of *Ae. aegypti* were able to transmit the two ZIKV isolates of the Asian genotype at 14 and/or 21 d.p.i, while the sympatric *Ae. albopictus* were often unable to transmit or transmitted with very low efficiency. No difference was detected when comparing viral load in positive salivas of sympatric *Ae. albopictus* and *Ae. aegypti* infected with the same ZIKV isolate (Tables S3–S5).

### 2.6. Culex quinquefasciatus from New Caledonia Is Refractory to ZIKV

No infection was observed for *Cx. quinquefasciatus* from New Caledonia, irrespective of the d.p.i. and the ZIKV strain tested (Table S1).

## 3. Discussion

We evaluated vector competence to three ZIKV lineages among 13 mosquito populations from Brazil and New Caledonia including *Ae. aegypti*, *Ae. albopictus* and *Cx. quinquefasciatus*. This study is unique in including a number of mosquito populations of three mosquito species from two continents and testing multiple ZIKV strains from three continents all under identical experimental conditions. The two territories focused on, i.e., Brazil and New Caledonia, were at the origin of the recent ZIVK pandemic.

With the exception of *Cx. quinquefasciatus,* all challenged New Caledonian and Brazilian *Aedes* were susceptible to infection with all tested ZIKV strains. However, viral dissemination to secondary tissues and transmission was highly variable among the 12 *Aedes* populations; ZIVK lineage and incubation time strongly influenced differences. The African ZIKV disseminated faster and at higher rates, regardless of incubation time in all 12 *Aedes* populations, compared to the Asian and American ZIKV lineages with a single exception, i.e., Kone *Ae. aegypti* at 21 d.p.i. The decrease in susceptibility to infection in these mosquitoes suggests that *Ae*. *aegypti* from Kone could be more resistant to infection by African ZIKV than those from Noumea. The African strain was the only one to have been transmitted as early as 7 d.p.i. in both *Ae. aegypti* and *Ae. albopictus,* with the two New Caledonian *Ae. aegypti* populations being significantly more efficient in transmitting this strain than the Brazilian populations. These advantages in transmission rates of the African ZIKV genotype relative to the Asian genotype were similarly described for Guadalupian *Ae. aegypti*, challenged with the same isolates and viral load [39]. Previous studies with other African ZIKV strains have shown greater transmission rates in *Ae. aegypti* from Mexico, USA, Brazil and New Caledonia, as well as higher fitness in vitro compared to ZIKV of the Asian genotype [8,31,40,41]. Curiously, it has been shown that New Caledonian *Ae. aegypti* are able to transmit African ZIKV isolates earlier than both the American and Asian ZIKV isolates [42]. It is worth noting that all seven New Caledonian and Brazilian *Ae. aegypti* tested here transmitted all virus lineages from two weeks or more after oral challenge, with the viral load in positive Brazilian *Ae. aegypti* salivas not differing among the tested viruses, except for those from Rio de Janeiro, which presented higher viral loads when infected with the African isolate compared to the American one at 14 d.p.i. When considering the performance of the two ZIKV strains belonging to the Asian genotype in *Ae. aegypti*, we found that the dissemination rates of the Asian lineage were lower than those detected for the American lineage, independent of the mosquito population origin, while rates of transmission did not differ. Interestingly, the seven Brazilian and New Caledonian *Ae. aegypti* did not differ in their efficiency of transmitting the American ZKV lineage at 14 and 21 days’ extrinsic incubation. The American ZIKV lineage, the only one circulating in Brazil, has caused disease outbreaks with distinct incidences in the five Brazilian regions that were the origin of *Ae. aegypti* populations assessed herein. During the 2016 outbreak, the Central-western region had the highest incidence of probable Zika cases (222.0/100 thousand inhabitants), followed by the Northeast (134.4/100 thousand inhab.), Southeast (106.2/100 thousand inhab.) and South (3.4/100 thousand inhab.) [12]. Annual incidences in all regions have decreased considerably in recent years, but the Northeast continues to report the highest rates (9.5/100 thousand inhab. in 2019) [14]. In this context, it is important to point out that vector competence is only one component determining the vectorial capacity of *Ae. aegypti* populations, the accurate assessment of which requires analyses of epidemiological, environmental and climate factors to help better understand the vector transmission dynamics and extent and intensity of urban arbovirus transmission [20,43,44].

The vector competence of Brazilian *Ae. albopictus* has been poorly evaluated [35,45]. Here, we assessed the vector competence of sympatric *Ae. aegypti* and *Ae. albopictus* from five Brazilian cities. In agreement with previous works [25,35,46,47], *Ae. aegypti* displayed greater susceptibility to infection and likelihood of transmitting the three tested ZIKVs compared to *Ae. albopictus* from Brazil. In the present study, the dissemination rates and transmission efficiency in *Ae. aegypti* were significantly higher than in *Ae. albopictus*, especially for the African ZIKV, although this isolate could be detected in the saliva of all five tested *Ae. albopictus* populations. Ciota et al. (2017) [48] found that USA *Ae. albopictus* were more susceptible to infection than *Ae. aegypti*, but that transmission efficiency was lower than *Ae. aegypti,* a result that suggested the existence of important transmission barriers in *Ae. albopictus.* In contrast to the African ZIKV, Brazilian *Ae. albopictus* were incapable of transmitting ZIKV of the Asian and American lineages in essentially all cases. The inefficiency in transmitting American strains of ZIKV was previously reported [45]. Importantly, in spite of its potential to transmit ZIKV, including reports of natural ZIKV infections elsewhere, there is no solid evidence that *Ae. albopictus* has caused human ZIKV transmission in Brazil [20,21,23,25].

The epidemiological histories of ZIKV differ in Brazil and New Caledonia, but they are quite related by the fact that the rapid spread of the virus in Brazil was preceded by the epidemic passage of the same genotype in the Pacific, particular in New Caledonia [49,50]. On the other hand, the histories of colonization by the two *Aedes* species tested herein differed considerably. While *Ae. aegypti* is very widespread in Brazil as well as in most of the Pacific islands, the invasive Asian mosquito *Ae. albopictus* has not yet colonized New Caledonia [51]. The invasion and colonization of the Americas by the African mosquito *Ae. aegypti* dates back more than four centuries; the species was eradicated in Brazil in the 1950s and later reintroduced from populations from neighboring countries [52,53,54], while the Pacific area and New Caledonia were colonized more recently, mostly at the end of the XIXth and beginning of the XXth century by founders of Asian, Australian and American origins [51,55]. In addition, the establishment of populations in distinct ecosystems, climates and environments, as well as exposure to control measures, differently influenced natural history, microbiota and population genetics, which, in turn, modulated phenotypes like vector competence [38,56].

The Brazilian mosquito populations tested here originated from five cities with differing demographic and infrastructural conditions, as well as being environmentally distinct, varying from semiarid to equatorial, with primary vegetation coverage from the savanna-like *cerrado* to the Atlantic and Amazon rain forest. The relatively low human population densities of the inland cities of Cuiabá, Manaus and Londrina (163.88, 158.06 and 306.49 inhabitants/Km^2^, respectively) contrast with high densities in the coastal Natal and Rio de Janeiro (4808.20 and 5265.81 inhab./Km^2^) [57]. Insecticide-induced changes in resistant mosquitoes may be associated with phenotypic variations in vector competence, either through sublethal exposure to insecticides or indirectly through changes in the environment [56]. Genes controlling resistance to insecticides can have pleiotropic effects and result in changes in the insect’s vector capacity, such as longevity, behavior, and vector competence [58]. The Brazilian *Aedes* mosquito populations tested here differed considerably in terms of their resistance to insecticides such as Temephos and Deltramethrin which were employed in the National Dengue Control Program [59,60,61,62,63,64,65,66]. The Natal and Rio de Janeiro *Ae. aegypti* populations exhibit greater resistance rates to the larvicide Temephos (an organophosphate), while Londrina and Manaus exhibit higher resistance to the adulticide Deltramethrin (a pyrethroid) [65]. The high frequency of *kdr* alleles that contribute to pyrethroid resistance is widespread in Brazilian *Ae. aegypti*, including the populations challenged here [65], and the Phe1534Cys *kdr* mutation has been detected in *Ae. albopictus* populations, including in the nearby of Londrina [64]. In New Caledonia, important resistant *kdr* mutations were not detected in Noumea *Ae. aegypti*, which display low resistance levels to Deltramethrin [67]. Large genetic differentiation has been reported among Brazilian *Ae. aegypti* [68,69,70,71,72]. In contrast, lower genetic differentiation has been recorded between New Caledonian *Ae. aegypti* from the main island (Noumea and Poindimie) than from a smaller island (from Ouvea) [51]. Additionally, except for differences in demographic densities, Noumea (2186.6 inhabitants/Km^2^) and Kone (19.6 inhabitants/Km^2^) are quite similar with regard to climate (http://www.meteo.nc/nouvelle-caledonie/climat/fiches-climatologiques). Together, these observations help to explain similarities in the infection and dissemination rates between the two New Caledonian *Ae. aegypti* populations, regardless of the tested viruses. However, transmission rates and efficiency differed, which may suggest different frequencies in transmission barriers. In sum, heterogeneity in vector competence to different ZIKV lineages in *Ae. aegypti* and *Ae. albopictus* is not surprising, considering their differing origins and histories [20,23,45].

As expected, the New Caledonian *Cx. quinquefasciatus* was refractory to all three ZIKV strains. This result agrees with the repeated vector competence evaluations performed with ZIKV isolates and *Cx. quinquefasciatus*, as well as *Cx. pipiens* from several origins [28,30,40,48,73,74,75,76,77,78,79,80,81]. In view of these data, no role in the human transmission of ZIKV can be attributed to *Cx. quinquefasciatus* from New Caledonian or elsewhere [30,74,81].

There are likely genetic, technical and environmental factors that contribute to the variations in vector competence in *Aedes* mosquitoes observed in the laboratory that may not always be relevant to phenotypic variations in nature [38,40,45,82,83]. For example, mosquitoes fed directly on viremic ZIKV vertebrates tend to become more infected and transmit better. Thus, it is possible that Brazilian and New Caledonian *Aedes* are more competent than what we observed in the laboratory, which indirectly suggests a potentially greater risk of transmission. While population origin influences transmission efficiency, Brazilian and New Caledonian *Ae. aegypti* are competent vectors for ZIKV of African, Asian and American lineages, with advantages for the African lineage, which can also be transmitted by Brazilian *Ae. albopictus.* Reinforcing viral surveillance and improving and strengthening control measures to reduce infestation by domestic and peridomestic *Aedes* are imperative if endemic Brazilian and New Caledonian territories are to avoid the spread of new ZIKV lineages and mitigate the transmission of locally circulating ZIKV in Brazil.

## 4. Materials and Methods

### 4.1. Ethic Statements

This study was done in compliance with New Caledonia Ethic regulations and the Institutional Ethics Committee on Animal Use (CEUA-IOC license LW-34/14) at the Instituto Oswaldo Cruz. This study did not involve endangered or protected species.

### 4.2. Viral Strains

Three ZIKV strains were used, each representing a viral lineage: DAK 84 (Senegal 1984, African genotype), MRS_OPY_Martinique_PaRi_2015 (Martinique 2015, Asian genotype, American lineage) and MASS 66 (Malaysia 1966, Asian genotype, Asian lineage). Viruses were provided by the Emergence Virus Unit (Marseilles, France) via the European EVAg project. Lyophilizates were resuspended in sterile distilled water and inoculated onto Vero cells (ATCC, ref. CCL-81) for viral production using a multiplicity of infection of 0.1 and DMEM medium supplemented with 2% fetal bovine serum (FBS). Supernatants were collected after three days of growth and stored at −80 °C prior to vector competence experiments. The viral titer of each virus strain was determined by serial 10-fold dilutions of viral stock on Vero cells, and was expressed as 50% tissue culture infective dose per milliliter (TCID_50_/mL). In the case of MRS_OPY_Martinique_PaRi_2015 ZIKV strain, virus stock was centrifuged using Vivaspin 6 centrifugal concentrator (Sartorius, Stonehouse, UK) to achieve the final concentration at 10^7^ TCID_50_/mL used in the oral challenge experiments.

### 4.3. Mosquito Populations

**Figure 5:**
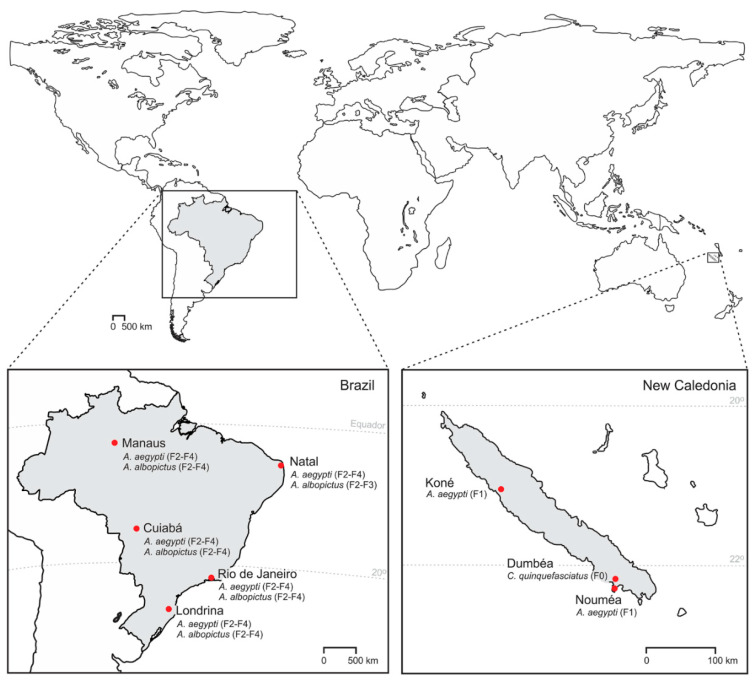
Species and localization of mosquitoes from Brazil and New Caledonia challenged with Zika virus. The generations of tested mosquitoes are in parenthesis.

Three mosquito species of eight populations were orally challenged: two of *Ae. aegypti* (F1 generation) and one of *Cx. quinquefasciatus* (F0 generation) from New Caledonia, and five of *Ae. aegypti* (F2–F4 generation) and *Ae. albopictus* (F2–F4 generation) from Brazil (Figure 5). In New Caledonia, *Ae. aegypti* (larvae) and *Cx. quinquefasciatus* (eggs rafts) were collected in Noumea (Southwestern), Kone (Northwestern) and Dumbea (Southwestern) during the hot season (April and March 2018) (Figure 1). The Brazilian *Ae. aegypti* and *Ae. albopictus* were derived from eggs collected with ovitraps set in Manaus (Northern, Amazon), Natal (Northeastern), Rio de Janeiro (Southeastern), Cuiabá (West-Central), Londrina (Southern) at different periods (Figure 5). Larvae were reared in 2 L plastic pans, with a density of 150–200 larvae per pan containing dechlorinated tap water, supplemented with brewer’s yeast which was renewed every 2–3 days. Pupae were then transferred to rearing cages where adults were maintained at 28 ± 1 °C, 70–80% relative humidity and a 12:12 h light:dark cycle, with access to 10% sucrose solution ad libitum.

### 4.4. Oral Challenge

Seven- to ten-day-old nulliparous females were used for oral infection, with a preliminary starvation of 24 h and 48 h for *Aedes* species and *Cx. quinquefasciatus,* respectively. The infectious blood meal consisted of a mix (2:1) of washed rabbit erythrocytes and viral suspension at 3 × 10^7^ TCID_50_/mL (final concentration at 10^7^ TCID_50_/mL) supplemented with a phagostimulant (5 mM ATP). Mosquito feeding was performed for 20 min with a Hemotek system (Hemotek Limited, Great Harwood, UK). Fully engorged females were transferred into cardboard containers and maintained with 10% sucrose solution at 28 ± 1 °C, 70–80% relative humidity and a 12:12 h light:dark cycle for further analysis.

### 4.5. Infection, Dissemination and Transmission Analyses

For each combination of viral strain and mosquito population, 20–30 females were randomly analyzed at 7, 14 and 21 days after oral challenge (hereinafter abbreviated as d.p.i.). Mosquitoes were individually processed using disposable and disinfected supplies to avoid contamination between individuals and between tissues of the same mosquito, as previously described [84]. After brief anesthesia by exposure to cold (ice bath) and removal of the wings and legs, mosquito saliva was collected in 5 µL of FBS for 30 min at room temperature. Each saliva sample was added to 45 µL of DMEM medium and stored at −80 °C until use. For the determination of viral infection and dissemination, each mosquito body (abdomen and thorax) and head was separately ground in 350 µL of DMEM medium supplemented with 2% FBS and antibiotics/antifungals (100 units/mL of penicillin, 0.1 mg/mL of streptomycin and 0.25 µg/mL amphotericin B). The obtained homogenates were then centrifuged at 10,000 *g* for 5 min at 4 °C before inoculation in cell culture. The infectious status was determinate by the presence of cytopathogenic effect (CPE). Briefly, 100 µL of diluted samples were inoculated onto confluent monolayer Vero cells in 96-well plates, and incubated for 7 days at 37 °C with 5% CO_2_ under 2.4% CMC (carboxymethyl cellulose) or agarose. Plates were then stained with a 0.2% crystal violet solution (in 10% formaldehyde and 20% ethanol). The presence of ZIKV particles in saliva and viral titer were determined by plaque assay. Saliva samples were inoculated in six-well plates seeded with Vero cells, and incubated and stained as described above. Saliva titers were expressed as plaque forming units (PFU) per saliva sample. The infection rate corresponded to the proportion of mosquitoes with infected bodies among all the tested mosquitoes. The dissemination rate represented the proportion of mosquitoes with infectious heads among infected mosquitoes. The transmission rate corresponded to the proportion of mosquitoes with infectious viral particles in saliva among mosquitoes with infected heads. The transmission efficiency corresponded to the proportion of mosquitoes with virus in saliva among all the orally challenged mosquitoes.

### 4.6. Statistical Analyses

The different rates and efficiencies calculated either by mosquito populations, viral strains or days postchallenge were compared by a Fisher’s exact test. Quantitative data corresponding to viral titer per saliva were compared using Wilcoxon test. All tests were corrected for multiple comparisons using the Holm method. All statistical analyses were performed with the R v3.6.1 software [85] considering *p-*values < 0.05 as significant.

## Supplementary Materials

The following are available online at https://www.mdpi.com/2076-0817/9/7/575/s1, Table S1: Infection rates, dissemination rates, transmission rates and transmission efficiencies obtained for all the mosquito populations tested with the three Zika virus isolates at 7, 14 and 21 days after oral challenge. Table S2: Medians and interquartile rages of viral load in saliva of *Aedes aegypti* Brazilian populations challenged with the three Zika virus isolates at 7, 14 and 21 days after challenge. Table S3: Summarize of the *p*-values obtained with the Fisher’Exact test and the Wilcoxon test corrected with the Holm method of infection rates, dissemination rates, transmission rates, transmission efficiencies and viral loads in saliva obtained for all the mosquito populations tested with the Zika virus of the African lineage at 7, 14 and 21 days after oral challenge. Table S4: Summarize of the *p*-values obtained with the Fisher’Exact test and the Wilcoxon test corrected with the Holm method of infection rates, dissemination rates, transmission rates, transmission efficiencies and viral loads in saliva obtained for all the mosquito populations tested with the Zika virus of the Asian lineage at 7, 14 and 21 days after oral challenge. Table S5: Summarize of the *p*-values obtained with the Fisher’Exact test and the Wilcoxon test corrected with the Holm method of infection rates, dissemination rates, transmission rates, transmission efficiencies and viral loads in saliva obtained for all the mosquito populations tested with the Zika virus of the American lineage at 7, 14 and 21 days after oral challenge. Table S6: Medians and interquartile rages of viral load in saliva of *Aedes albopictus* Brazilian populations challenged with the three Zika virus isolates at 7, 14 and 21 days after challenge.
