# Supplementary material for: Vector Competence of Aedes aegypti, Aedes albopictus and Culex quinquefasciatus from Brazil and New Caledonia for Three Zika Virus Lineages
Source: Pathogens. 2020 Jul 16;9(7):575. doi: 10.3390/pathogens9070575 (PMC7399907; doi:10.3390/pathogens9070575)
Supplement: Supplementary file 1 [file pathogens-09-00575-s001.zip › Suppl file - S2 table - Viral loads saliva Ae aegypti.docx]

**Table S2. Medians and interquartile rags of viral load in saliva of *Aedes aegypti* Brazilian populations challenged with the three Zika virus isolates at 7, 14 and 21 days after challenge.**

| **Days** | **Mosquito population** | | | **Virus** | **Number**  **of positive salivas** | | **Median*** | **Interquartile range*** |
| --- | --- | --- | --- | --- | --- | --- | --- | --- |
| 7 | Cuiabá | | | DAK 84 | 0 | | - | - |
|  |  |  |  | MASS 66 | 0 | | - | - |
|  |  |  |  | MRS OPY | 0 | | - | - |
|  | Londrina | | | DAK 84 | 2 | | 2 | [2 ; 2] |
|  |  |  |  | MASS 66 | 0 | | - | - |
|  |  |  |  | MRS OPY | 0 | | - | - |
|  | Manaus | | | DAK 84 | 5 | | 36 | [33 ; 241] |
|  |  |  |  | MASS 66 | 0 | | - | - |
|  |  |  |  | MRS OPY | 0 | | - | - |
|  | Natal | | | DAK 84 | 2 | | 36 | [32 ; 40] |
|  |  |  |  | MASS 66 | 0 | | - | - |
|  |  |  |  | MRS OPY | 0 | | - | - |
|  | Rio de Janeiro | | | DAK 84 | 5 | | 13 | [2 ; 38] |
|  |  |  |  | MASS 66 | 0 | | - | - |
|  |  |  |  | MRS OPY | 0 | | - | - |
| 14 | Cuiabá | | | DAK 84 | 26 | | 94.50 | [23.75 ; 161.25] |
|  |  |  |  | MASS 66 | 0 | | - | - |
|  |  |  |  | MRS OPY | 3 | | 114 | [59 ; 118.5] |
|  | Londrina | | | DAK 84 | 24 | | 29.50 | [9 ; 105] |
|  |  |  |  | MASS 66 | 1 | | 2 | [2 ; 2] |
|  |  |  |  | MRS OPY | 5 | | 20 | [8 ; 49] |
|  | Manaus | | | DAK 84 | 24 | | 19.50 | [7.75 ; 106.50] |
|  |  |  |  | MASS 66 | 3 | | 38 | [24.50 ; 39.50] |
|  |  |  |  | MRS OPY | 3 | | 9 | [6 ; 51] |
|  | Natal | | | DAK 84 | 20 | | 54.50 | [12.25 ; 154.50] |
|  |  |  |  | MASS 66 | 2 | | 160.50 | [125.80 ; 195.20] |
|  |  |  |  | MRS OPY | 4 | | 53 | [23.75 ; 110.75] |
|  | Rio de Janeiro | | | DAK 84 | 27 | | 49 | [24 ; 124.50] |
|  |  |  |  | MASS 66 | 0 | | - | - |
|  |  |  |  | MRS OPY | 6 | | 16.50 | [13.25 ; 19.75] |
| 21 | Cuiabá | | | DAK 84 | 21 | | 36 | [7 ; 98] |
|  |  |  |  | MASS 66 | 3 | | 23 | [19.50 ; 76] |
|  |  |  |  | MRS OPY | 5 | | 12 | [9 ; 14] |
|  | Londrina | | | DAK 84 | 24 | | 21 | [11.75 ; 54.25] |
|  |  |  |  | MASS 66 | 1 | | 2 | [2 ; 2] |
|  |  |  |  | MRS OPY | 2 | | 97 | [59 ; 135] |
|  | Manaus | | | DAK 84 | 21 | | 25 | [8 ; 40] |
|  |  |  |  | MASS 66 | 0 | | - | - |
|  |  |  |  | MRS OPY | 8 | | 50.50 | [21 ; 125.20] |
|  | Natal | | | DAK 84 | 26 | | 66 | [38.50 ; 120] |
|  |  |  |  | MASS 66 | 1 | | 90 | [90 ; 90] |
|  |  |  |  | MRS OPY | NT | | - | - |
|  | Rio de Janeiro | | | DAK 84 | 20 | | 12 | [3.75 ; 20.50] |
|  |  |  |  | MASS 66 | 2 | | 7.50 | [7.25 ; 7.75] |
|  |  |  |  | MRS OPY | 1 | | 14 | [14 ; 14] |
|  | |  |  | | |  |  |  |

* Medians and interquartile ranges are expressed in PFU per saliva

NT: Not Tested; ZIKV isolates: DAK 84 (African lineage), MASS 66 (Asian lineage), MRS OPY (American lineage).
