# Supplementary material for: Vector Competence of Aedes aegypti, Aedes albopictus and Culex quinquefasciatus from Brazil and New Caledonia for Three Zika Virus Lineages
Source: Pathogens. 2020 Jul 16;9(7):575. doi: 10.3390/pathogens9070575 (PMC7399907; doi:10.3390/pathogens9070575)
Supplement: Supplementary file 1 [file pathogens-09-00575-s001.zip › Suppl file - S6 table - Viral titers saliva Ae albopictus.docx]

**Table S3. Medians and interquartile rags of viral load in saliva of *Aedes albopictus* Brazilian populations challenged with the three Zika virus isolates at 7, 14 and 21 days after challenge.**

| **Day after challenge** | **Mosquito population** | **Virus** | **Number**  **of positive salivas** | **Median*** | **Interquartile range*** |
| --- | --- | --- | --- | --- | --- |
| 7 | Cuiabá | DAK 84 | 0 | - | - |
|  |  | MASS 66 | 0 | - | - |
|  |  | MRS OPY | 0 | - | - |
|  | Londrina | DAK 84 | 4 | 2 | [2 ; 3] |
|  |  | MASS 66 | 0 | - | - |
|  |  | MRS OPY | 0 | - | - |
|  | Manaus | DAK 84 | 0 | - | - |
|  |  | MASS 66 | 0 | - | - |
|  |  | MRS OPY | NT | - | - |
|  | Natal | DAK 84 | 0 | - | - |
|  |  | MASS 66 | NT | - | - |
|  |  | MRS OPY | NT | - | - |
|  | Rio de Janeiro | DAK 84 | 0 | - | - |
|  |  | MASS 66 | 0 | - | - |
|  |  | MRS OPY | 0 | - | - |
| 14 | Cuiabá | DAK 84 | 10 | 21 | [4 ; 189.80] |
|  |  | MASS 66 | 0 | - | - |
|  |  | MRS OPY | 0 | - | - |
|  | Londrina | DAK 84 | 15 | 80 | [41.50 ; 89.50] |
|  |  | MASS 66 | 3 | 22 | [14.50 ; 74.50] |
|  |  | MRS OPY | 0 | - | - |
|  | Manaus | DAK 84 | 10 | 6.50 | [4.50 ; 37.50] |
|  |  | MASS 66 | 0 | - | - |
|  |  | MRS OPY | 0 | - | - |
|  | Natal | DAK 84 | 0 | - | - |
|  |  | MASS 66 | NT | - | - |
|  |  | MRS OPY | 10 | 32 | [12.25 ; 86.25] |
|  | Rio de Janeiro | DAK 84 | 12 | 36.50 | [21.75 ; 58.25] |
|  |  | MASS 66 | 0 | - | - |
|  |  | MRS OPY | 0 | - | - |
| 21 | Cuiabá | DAK 84 | 18 | 51.50 | [16.75 ; 148.75] |
|  |  | MASS 66 | 0 | - | - |
|  |  | MRS OPY | NT | - | - |
|  | Londrina | DAK 84 | 13 | 27 | [9 ; 84] |
|  |  | MASS 66 | 2 | 47.50 | [24.25 ; 70.75] |
|  |  | MRS OPY | 0 | - | - |
|  | Manaus | DAK 84 | 13 | 60 | [28 ; 104] |
|  |  | MASS 66 | 0 | - | - |
|  |  | MRS OPY | NT | - | - |
|  | Natal | DAK 84 | 11 | 44 | [20.50 ; 119] |
|  |  | MASS 66 | NT | - | - |
|  |  | MRS OPY | NT | - | - |
|  | Rio de Janeiro | DAK 84 | 17 | 27 | [14 ; 172] |
|  |  | MASS 66 | 1 | 3 | [3 ; 3] |
|  |  | MRS OPY | 0 | - | - |

* Medians and interquartile ranges are expressed in PFU per saliva

NT: Not Teste; ZIKV isolates: DAK 84 (African lineage), MASS 66 (Asian lineage), MRS OPY (American lineage).
