# Supplementary material for: Vector Competence of Aedes aegypti, Aedes albopictus and Culex quinquefasciatus from Brazil and New Caledonia for Three Zika Virus Lineages
Source: Pathogens. 2020 Jul 16;9(7):575. doi: 10.3390/pathogens9070575 (PMC7399907; doi:10.3390/pathogens9070575)
Supplement: Supplementary file 1 [file pathogens-09-00575-s001.zip › Supplementary file - S1 Table .docx]

**S1 Table. Infection rates, dissemination rates, transmission rates and transmission efficiencies obtained for all the mosquito populations tested with the three Zika virus isolates at 7, 14 and 21 days after challenge.**

| **Mosquito species** | **Day after challenge** | **Mosquito population** | **Infection rate (%)** | | | **Dissemination rate (%)** | | | | **Transmission rate (%)** | | | | **Transmission efficiency (%)** | | | |
| --- | --- | --- | --- | --- | --- | --- | --- | --- | --- | --- | --- | --- | --- | --- | --- | --- | --- |
|  |  |  | **Dak 84** | **MASS 66** | **MRS OPY** | **Dak 84** | **MASS 66** | **MRS OPY** | **Dak 84** | | **MASS 66** | **MRS OPY** | **Dak 84** | | **MASS 66** | **MRS OPY** |  |
| *Aedes aegypti* | 7 | Cuiabà | 97 (29/30) | 70 (14/20) | 100 (20/20) | 45 (13/29) | 0 (0/14) | 10 (2/20) | 0 (0/13) | | NA | 0 (0/2) | 0 (0/30) | | 0 (0/20) | 0 (0/20) |  |
|  |  | Londrina | 100 (30/30) | 75 (15/20) | 77 (23/30) | 63 (19/30) | 0 (0/15) | 9 (2/23) | 11 (2/19) | | NA | 0 (0/2) | 7 (2/30) | | 0 (0/20) | 0 (0/30) |  |
|  |  | Manaus | 100 (30/30) | 30 (6/20) | 97 (29/30) | 90 (27/30) | 0 (0/6) | 0 (0/29) | 19 (5/27) | | NA | NA | 17 (5/30) | | 0 (0/20) | 0 (0/30) |  |
|  |  | Natal | 90 (27/30) | 60 (12/20) | 95 (19/20) | 85 (23/27) | 0 (0/12) | 21 (4/19) | 9 (2/23) | | NA | 0 (0/4) | 7 (2/30) | | 0 (0/20) | 0 (0/20) |  |
|  |  | Rio de Janeiro | 100 (30/30) | 25 (5/20) | 60 (18/30) | 93 (28/30) | 0 (0/5) | 0 (0/18) | 18 (5/28) | | NA | NA | 17 (5/30) | | 0 (0/20) | 0 (0/30) |  |
|  |  | Koné | 89 (25/28) | 63 (19/30) | 62 (18/29) | 100 (25/25) | 79 (15/19) | 72 (13/18) | 64 (16/25) | | 7 (1/15) | 15 (2/13) | 57 (16/28) | | 3 (1/30) | 7 (2/29) |  |
|  |  | Nouméa | 100 (20/20) | 90 (18/20) | 90 (18/20) | 100 (20/20) | 67 (12/18) | 61 (11/18) | 85 (17/20) | | 0 (0/12) | 0 (0/11) | 85 (17/20) | | 0 (0/20) | 0 (0/20) |  |
|  | 14 | Cuiabà | 100 (30/30) | 87 (26/30) | 90 (27/30) | 100 (30/30) | 35 (9/26) | 85 (23/27) | 87 (26/30) | | 22 (2/9) | 13 (3/23) | 87 (26/30) | | 7 (2/30) | 10 (3/30) |  |
|  |  | Londrina | 100 (30/30) | 93 (28/30) | 93 (28/30) | 97 (29/30) | 39 (11/28) | 79 (22/28) | 90 (26/29) | | 27 (3/11) | 23 (5/22) | 87 (26/30) | | 10 (3/30) | 17 (5/30) |  |
|  |  | Manaus | 100 (30/30) | 93 (28/30) | 87 (26/30) | 100 (30/30) | 61 (17/28) | 85 (22/26) | 90 (27/30) | | 35 (6/17) | 23 (5/22) | 90 (27/30) | | 20 (6/30) | 17 (5/30) |  |
|  |  | Natal | 73 (22/30) | 60 (18/30) | 87 (26/30) | 100 (22/22) | 22 (4/18) | 100 (26/26) | 95 (21/22) | | 50 (2/4) | 15 (4/26) | 70 (21/30) | | 7 (2/30) | 13 (4/30) |  |
|  |  | Rio de Janeiro | 97 (29/30) | 93 (28/30) | 67 (20/30) | 97 (28/29) | 25 (7/28) | 95 (19/20) | 96 (27/28) | | 14 (1/7) | 37 (7/19) | 90 (27/30) | | 3 (1/30) | 23 (7/30) |  |
|  |  | Koné | 100 (26/26) | 90 (26/29) | 96 (27/28) | 85 (22/26) | 92 (24/26) | 89 (24/27) | 82 (18/22) | | 67 (16/24) | 17 (4/24) | 69 (18/26) | | 55 (16/29) | 14 (4/28) |  |
|  |  | Nouméa | 100 (19/19) | 100 (21/21) | 85 (17/20) | 100 (19/19) | 86 (18/21) | 94 (16/17) | 100 (19/19) | | 33 (6/18) | 25 (4/16) | 100 (19/19) | | 29 (6/21) | 20 (4/20) |  |
|  | 21 | Cuiabà | 100 (30/30) | 73 (22/30) | 91 (20/22) | 100 (30/30) | 64 (14/22) | 100 (20/20) | 87 (26/30) | | 36 (5/14) | 25 (5/20) | 87 (26/30) | | 17 (5/30) | 23 (5/22) |  |
|  |  | Londrina | 100 (30/30) | 83 (5/6) | 87 (26/30) | 100 (30/30) | 60 (3/5) | 77 (20/26) | 80 (24/30) | | 33 (1/3) | 35 (7/20) | 80 (24/30) | | 17 (1/6) | 23 (7/30) |  |
|  |  | Manaus | 100 (30/30) | 94 (16/17) | 97 (29/30) | 100 (30/30) | 19 (3/16) | 93 (27/29) | 77 (23/30) | | 0 (0/3) | 37 (10/27) | 77 (23/30) | | 0 (0/17) | 33 (10/30) |  |
|  |  | Natal | 93 (28/30) | 69 (9/13) | NT | 100 (28/28) | 67 (6/9) | NT | 96 (27/28) | | 17 (1/6) | NT | 90 (27/30) | | 8 (1/13) | NT |  |
|  |  | Rio de Janeiro | 100 (30/30) | 73 (22/30) | 45 (5/11) | 100 (30/30) | 23 (5/22) | 60 (3/5) | 77 (23/30) | | 40 (2/5) | 33 (1/3) | 77 (23/30) | | 7 (2/30) | 9 (1/11) |  |
|  |  | Koné | 100 (23/23) | 100 (29/29) | 83 (20/24) | 100 (23/23) | 100 (29/29) | 100 (20/20) | 22 (5/23) | | 41 (12/29) | 25 (5/20) | 22 (5/23) | | 41 (12/29) | 21 (5/24) |  |
|  |  | Nouméa | 100 (30/30) | 95 (19/20) | 84 (21/25) | 97 (29/30) | 100 (19/19) | 95 (20/21) | 100 (29/29) | | 21 (4/19) | 50 (10/20) | 97 (29/30) | | 20 (4/20) | 40 (10/25) |  |
| *Aedes albopictus* | 7 | Cuiabà | 60 (18/30) | 23 (7/30) | 23 (7/30) | 39 (7/18) | 14 (1/7) | 0 (0/7) | 0 (0/7) | | 0 (0/1) | NA | 0 (0/30) | | 0 (0/30) | 0 (0/30) |  |
|  |  | Londrina | 57 (17/30) | 33 (10/30) | 17 (5/30) | 41 (7/17) | 10 (1/10) | 0 (0/5) | 57 (4/7) | | 0 (0/1) | NA | 13 (4/30) | | 0 (0/30) | 0 (0/30) |  |
|  |  | Manaus | 7 (2/30) | 32 (10/31) | NT | 50 (1/2) | 0 (0/10) | NT | 0 (0/1) | | NA | NT | 0 (0/30) | | 0 (0/31) | NT |  |
|  |  | Natal | 73 (22/30) | NT | NT | 0 (0/22) | NT | NT | NA | | NT | NT | 0 (0/30) | | NT | NT |  |
|  |  | Rio de Janeiro | 57 (17/30) | 13 (4/30) | 20 (6/30) | 41 (7/17) | 0 (0/4) | 17 (1/6) | 0 (0/7) | | NA | 0 (0/1) | 0 (0/30) | | 0 (0/30) | 0 (0/30) |  |
|  | 14 | Cuiabà | 77 (23/30) | 10 (3/30) | 23 (6/26) | 57 (13/23) | 0 (0/3) | 17 (1/6) | 77 (10/13) | | NA | 0 (0/1) | 33 (10/30) | | 0 (0/30) | 0 (0/26) |  |
|  |  | Londrina | 80 (24/30) | 30 (9/30) | 7 (2/30) | 83 (20/24) | 44 (4/9) | 0 (0/2) | 75 (15/20) | | 100 (4/4) | NA | 50 (15/30) | | 13 (4/30) | 0 (0/30) |  |
|  |  | Manaus | 70 (21/30) | 13 (4/30) | 27 (7/26) | 67 (14/21) | 0 (0/4) | 29 (2/7) | 79 (11/14) | | NA | 0 (0/2) | 37 (11/30) | | 0 (0/30) | 0 (0/26) |  |
|  |  | Natal | 77 (23/30) | NT | 83 (25/30) | 48 (11/23) | NT | 92 (23/25) | 0 (0/11) | | NT | 48 (11/23) | 0 (0/30) | | NT | 37 (11/30) |  |
|  |  | Rio de Janeiro | 60 (18/30) | 27 (8/30) | 30 (9/30) | 83 (15/18) | 38 (3/8) | 0 (0/9) | 80 (12/15) | | 0 (0/3) | NA | 40 (12/30) | | 0 (0/30) | 0 (0/30) |  |
|  | 21 | Cuiabà | 73 (22/30) | 43 (10/23) | NT | 91 (20/22) | 20 (2/10) | NT | 100 (20/20) | | 0 (0/2) | NT | 67 (20/30) | | 0 (0/23) | NT |  |
|  |  | Londrina | 53 (16/30) | 30 (9/30) | 17 (5/30) | 94 (15/16) | 67 (6/9) | 60 (3/5) | 87 (13/15) | | 17 (1/6) | 0 (0/3) | 43 (13/30) | | 3 (1/30) | 0 (0/30) |  |
|  |  | Manaus | 74 (20/27) | 33 (10/30) | NT | 90 (18/20) | 10 (1/10) | NT | 78 (14/18) | | 0 (0/1) | NT | 52 (14/27) | | 0 (0/30) | NT |  |
|  |  | Natal | 80 (24/30) | NT | NT | 75 (18/24) | NT | NT | 61 (11/18) | | NT | NT | 37 (11/30) | | NT | NT |  |
|  |  | Rio de Janeiro | 70 (21/30) | 7 (2/30) | 20 (6/30) | 100 (21/21) | 50 (1/2) | 83 (5/6) | 90 (19/21) | | 100 (1/1) | 0 (0/5) | 63 (19/30) | | 3 (1/30) | 0 (0/30) |  |
| *Culex quinquefasciatus* | 7 | Dumbéa | 0 (0/29) | 0 (0/30) | 0 (0/30) | NA | NA | NA | NA | | NA | NA | NA | | NA | NA |  |
|  | 14 | Dumbéa | 0 (0/30) | 0 (0/30) | 0 (0/30) | NA | NA | NA | NA | | NA | NA | NA | | NA | NA |  |
|  | 21 | Dumbéa | 0 (0/21) | 0 (0/20) | 0 (0/30) | NA | NA | NA | NA | | NA | NA | NA | | NA | NA |  |

ZIKV isolates: DAK 84 (African lineage), MASS 66 (Asian lineage), MRS OPY (American lineage).

NA : Not achievable ; NT: Not Tested

Infection rate (number of infected bodies / number of mosquitoes tested)

Dissemination rate (number of infected heads / number of infected bodies)

Transmission rate (number of infected saliva / number of infected heads)

Transmission efficiency (number of infected saliva / number of mosquitoes tested)
